# Direct observation of the dynamics of single metal ions at the interface with solids in aqueous solutions

**DOI:** 10.1038/srep43234

**Published:** 2017-02-23

**Authors:** Maria Ricci, William Trewby, Clodomiro Cafolla, Kislon Voïtchovsky

**Affiliations:** 1 University of Cambridge, Cavendish Laboratory, Cambridge CB3 0HE, UK; 2 Department of Physics, Durham University, Durham DH1 3LE, UK

## Abstract

The dynamics of ions adsorbed at the surface of immersed charged solids plays a central role in countless natural and industrial processes such as crystal growth, heterogeneous catalysis, electrochemistry, or biological function. Electrokinetic measurements typically distinguish between a so-called Stern layer of ions and water molecules directly adsorbed on to the solid’s surface, and a diffuse layer of ions further away from the surface. Dynamics within the Stern layer remain poorly understood, largely owing to a lack of *in-situ* atomic-level insights. Here we follow the dynamics of single Rb^+^ and H_3_O^+^ ions at the surface of mica in water using high-resolution atomic force microscopy with 25 ms resolution. Our results suggest that single hydrated Rb^+^ions reside τ_1_ = 104 ± 5 ms at a given location, but this is dependent on the hydration state of the surface which evolves on a slower timescale of τ_2_ = 610 ± 30 ms depending on H_3_O^+^ adsorption. Increasing the liquid’s temperature from 5 °C to 65 °C predictably decreases the apparent glassiness of the interfacial water, but no clear effect on the ions’ dynamics was observed, indicating a diffusion-dominated process. These timescales are remarkably slow for individual monovalent ions and could have important implications for interfacial processes in electrolytes.

Most solids are charged in aqueous solutions. The ions dissolved in the liquid tend to accumulate near the solid’s surface to ensure charge neutrality, forming a so-called electrical double layer. Countless natural and industrial processes rely on the organization and dynamics of ions in this layer, from crystal growth^1^,^2^,^3^, to heterogeneous catalysis^4^, electrochemistry^5^, or biological function^6^,^7^,^8^,^9^,^10^. Ionic distribution within the electrical double layer is typically described by the Gouy-Chapman-Stern model^11^ that comprises a Stern layer of largely static ions and water molecules adsorbed to the solid’s surface, and a diffuse layer of ions further away from the surface in the liquid. The diffuse layer region is generally well understood, but the Stern layer is more difficult to model, partly because continuous assumptions have to be made about an intrinsically discrete object where the size and nature of the adsorbed ions play an important role. Significantly, existing models assume a homogenous charge distribution within the Stern layer, which is unlikely to hold at the nanoscale. Recently, a handful of studies^12^,^13^,^14^,^15^,^16^ have shown that it is possible to observe *in-situ* single ions within the Stern layer using atomic force microscopy (AFM). However these investigations were conducted at equilibrium and no information about the ion’s dynamics could be derived. The motion of single ions at interfaces remains a central question in interfacial processes with significant implications in nanotechnology, for example in the development of novel power-generating devices^17^,^18^. Existing theories require corrections to explain experimental observations^19^,^20^,^21^. The ‘dynamic Stern model’, which describes electro-osmotic flow parallel to the surface, assumes that water molecules are immobile but ions can move within the Stern layer^22^,^23^,^24^. Current modelling tends to rely either on theoretical studies or on indirect and macroscopic measurements of mobility that rely on a large ensemble of ions^25^. At the present time very few methods can track individual ions at interfaces locally, and with suitable temporal resolution. Studies based on Scanning Electrochemical Microscopy^26^ or Polarisation Force Microscopy^27^ conducted in 95% controlled humidity suggest relaxation times (directly linked to the ionic mobility) between 20 and 30 ms for ions at the surface of calcite. Similar measurements found a relaxation time in the order of milliseconds for ions on mica in a relative humidity around 85%^28^. Increasing the humidity tends to increase the ions’ mobility but it is unclear how these measurements translate to solids fully immersed into water. Generally, experimental studies when available, suggest diffusion timescales in the order of few milliseconds for adsorbed ions in solution, but no direct measurements are available.

Theoretical studies based on molecular dynamics (MD) simulations showed that the decrease of diffusivity of Na^+^ and Ca^2+^ ions in close proximity to mica surface (first few Ångströms) was at most two orders of magnitude lower than the bulk diffusivity of the same ions^29^. Other MD studies reported higher average velocity of water molecules parallel to the surface plane^30^, highlighting the complex interplay between the behaviour of the water hydrating the adsorbed ions and the mica surface. The existence of a particular hydration landscape has been shown to affect the behaviour of adsorbed ions in a specific manner^13^. At the present time, most MD-base approaches are however limited to relatively short timescales (typically sub-microsecond) by the use of empirical potentials and limited computational power. Additionally, MD simulations cannot easily take into account water dissociation and pH effects, an important factor here. Such effects can be described by ab initio quantum mechanical approaches, but currently not over nanoseconds for systems involving thousands of molecules and ions.

Here we follow *in-situ* the dynamics of single metal ions at the surface of mica in water using high-resolution amplitude-modulation AFM with 25 ms resolution. Since water plays an important role in the organization and dynamics of adsorbed ions^13^,^15^, quantitative results are derived for Rb^+^ ions which exist mainly in a single hydration state^31^,^32^. Our approach allows us to follow simultaneously Rb^+^ and H_3_O^+^ ions, and discriminate instrumentation limitations. We show that singe hydrated Rb^+^ reside for τ_1_~100 ms at a given location, but this is dependent on the hydration state of the surface which evolves on a slower timescale of τ_2_~600 ms due to H_3_O^+^. Increasing the temperature appears to affect the behaviour of the hydration water at the surface of mica, but not the respective ions’ dynamics. These timescales, remarkably slow for individual monovalent ions, have important implications for many interfacial processes, for example in electrochemical and electrokinetic systems^20^,^33^,^34^, in self-assembly processes^35^,^36^, and at biointerfaces where ions modulate charge transport^6^,^7^,^8^,^9^,^10^, mechanical properties^37^,^38^,^39^,^40^, and function^6^.

## Results

**Figure 1:**
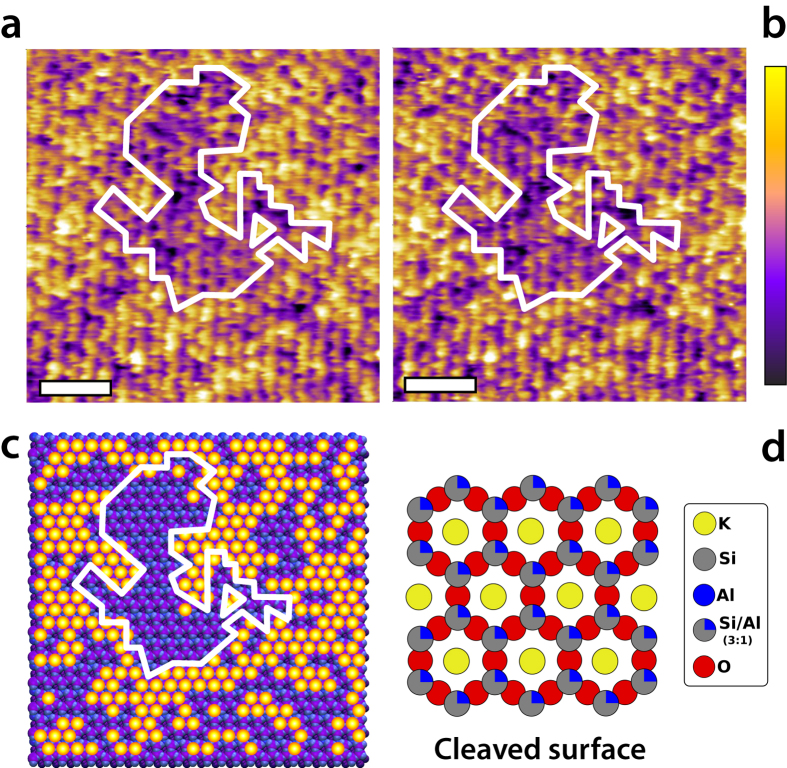
Topographic images of Rb^+^ ions adsorbed at the surface of mica in aqueous solution. The Rb^+^ ions appear yellow and the underlying mica surface purple. (**a**,**b**) were acquired at a same location with an average time interval of 45 ms. This was achieved by simultaneously recording an image with the tip scanning from right to left (**a**) and left to right (**b**) as it moves back. As a result, certain parts of the image experience shorter or longer time spans between two consecutive tip passes and 45 ms represents the average. A simplified cartoon representation of (**a**) is given (**c**), with a similar colour scale. The cartoon is to scale, with the surface of mica appearing purple and the adsorbed ions yellow. The detailed atomic structure of the exposed mica surface is shown in (**d**). In bulk mica, the centres of the hexagonal rings correspond to that of ditrigonal cavities that are occupied by K^+^ ions. When the mica is cleaved, the K^+^ ions are exposed on its surface. Here, the K^+^ ions have dissolved in the solution and are replaced by Rb^+^ ions that are present in much higher concentration and can adsorb at the same location. A gap in the Rb^+^ layer is highlighted with a white line in (**a–c**), showing little changes between (**a**,**b**). Images acquired subsequently exhibit a larger number of changes in the ionic layer, confirming that the adsorbed ions are mobile on longer timescales (shown in supplementary Fig. S1). The scale bar is 3 nm (**a,b**) and the colour scale represents 0.3 nm height variation.

In a typical AFM measurement of ions adsorbed at a solid-liquid interface, a nanometre-sharp tip is used to probe the interface locally and dynamically. The tip, mounted on an oscillating cantilever fully immersed into the solution, is brought in close vicinity to the interface. If the oscillation amplitude is small enough (typically <1 nm), local changes in the structure, density and dynamics of the hydration water can be detected as shifts in the amplitude, phase and frequency of the vibrating cantilever^41^,^42^,^43^,^44^. Adsorbed ions locally affect the hydration landscape of the solid and can hence be detected, often with angstrom resolution^12^,^13^,^15^,^45^,^46^,^47^. These ions generally appear in the topographic images as protruding features on the flat crystalline surface (bright “blobs” in Fig. 1a,b or in refs 12 and 13). The time resolution of such a measurement is limited by the vibration frequency of the cantilever. Here the frequency is typically 430 kHz in water, allowing the tip to probe the interface every 2.3 microseconds. However, integration over multiple oscillation cycles is required to achieve a reliable signal, and atomic-level images are typically limited to ~50 μs/pixel if to maintain sufficient resolution.

The AFM tip can in principle affect the measurements^13^,^48^,^49^,^50^,^51^,^52^ and it is hence necessary to move the tip several nanometres away from a given location of the interface before revisiting it so as to allow for local relaxation without the tip being present. Images of the interface acquired with an average time of 50 ms between two passes over the same location show adsorbed ions to be remarkably stable on the surface (Fig. 1), often visible at a same location for several seconds or even minutes. Water can play an important role in stabilizing ions at surfaces, and previous work has shown water-induced attractive correlation between adsorbed Rb^+^ ions on mica^13^. This is consistent with the result shown in Fig. 1 where domains of adsorbed Rb^+^ ions remain largely stable throughout the imaging process, with subsequent images revealing only limited changes near the edges of domains (see supplementary Fig. S1).

**Figure 2:**
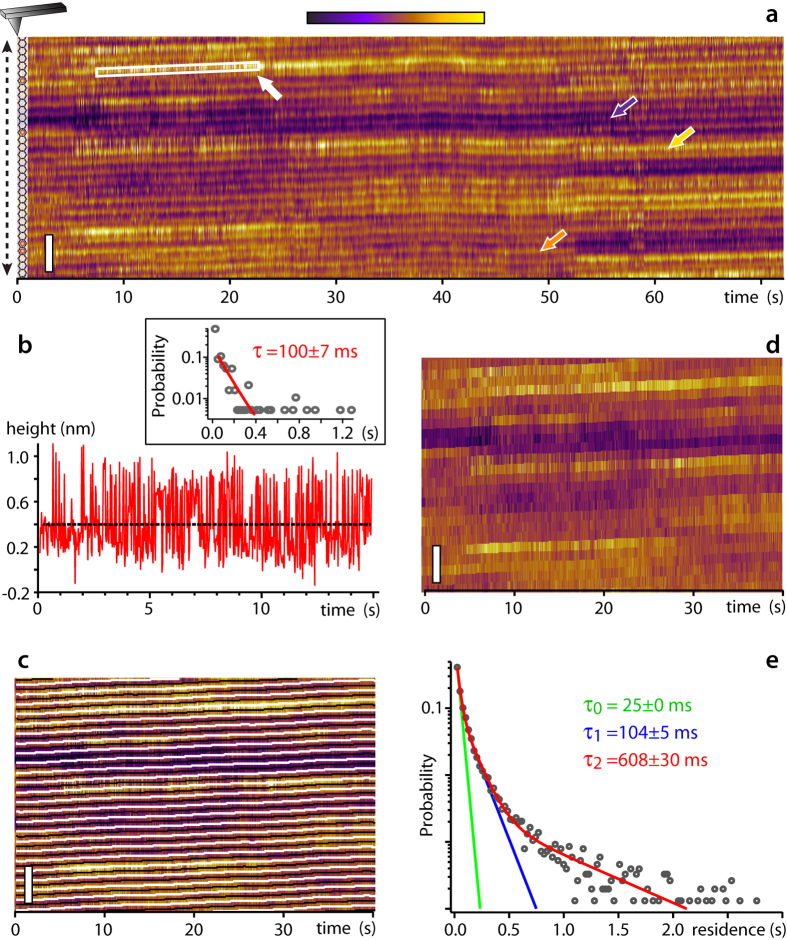
Time evolution of adsorbed Rb^+^ ions at the surface of mica in aqueous solution. The AFM tip repeatedly scans the same location along the (100) direction of the crystal at 40 lines/s (**a**). A cartoon representation of the scanned crystal surface with adsorbed ions is shown to scale on the left. The system is stable enough to follow the same atomic sites for minutes. Three distinct height levels can be identified, represented by coloured arrows (see supplementary Fig. S2). A profile taken over a selected site (white arrow in a) provides the time evolution of the apparent height over this site (**b**). Assuming that the height variations are induced by ions adsorbing/desorbing, thresholding analysis (dotted black line in **b**) derives a distribution of time intervals spent by ions on the site. Fitting of the distribution with a single exponential decay provides a timescale of typically 100 ms (inset in **b**). The residence probability is the normalized number of events. A homemade algorithm automatically tracked each site position with time, taking into account drift (**c**): white lines indicate the location of binding sites’ centre and the black lines the limit between two adjacent sites. Only a fraction of the image given in (**a**) is shown as an example. All the pixels associated with a given site at a given time (interval between two adjacent black lines) are then height-averaged thereby minimising the impact of mica corrugation and specific details of the tip hydration sites dominating the imaging^48^,^49^ (**d**). Profile analysis conducted over every site yielded (>than 30 min ×site) reveals three distinct timescales (**e**). First, a rapid timescale (25 ms) coincides with the scanning frequency and is imposed as a fitting parameter. The second and third timescale (104 ± 5 ms and 610 ± 30 ms) reflect real changes occurring over the sample (The threshold value in (**e**) is −0.1 nm, see Supplementary Figs S4–S7 for more details about the analysis). The data in (**e**) combines analysis from other experiments acquired in identical conditions (supplementary Fig. S9). The colour scale in (**a,c,d**) is 1.5 nm and the scale bar is 3 nm.

Although AFM images such as those presented in Fig. 1 qualitatively suggest residence times above 50 ms for adsorbed ions, a clear interpretation is not trivial. Previous AFM studies have explained the protrusions revealed by the AFM as ionic densities rather than single ions^14^,^53^. In order to better quantify the dynamics of these protrusions, we used the AFM to scan repeatedly over a same location of the surface spanning several ion-binding sites (Fig. 2). Practically, this is achieved by orienting the AFM’s fast scanning direction along one of the crystal axis of the mica while imaging a same profile at higher frequency. Here scanning was conducted at 40 Hz, thus providing an effective time resolution of 25 ms. The resulting images (for example Fig. 2a) show the topographic evolution of several unit cells of the crystal with time. Three distinct surface states are visible as three different height levels on the surface (arrows in Fig. 2a), all with a relatively slow dynamic (see height distribution analysis in Supplementary Fig. S2). We interpreted the state corresponding to the highest features on the surface (yellow arrow in Fig. 2a) as hydrated Rb^+^ ions absorbed above the ditrigonal cavity (see Fig. 1d) of the mica the surface^31^,^54^,^55^,^56^. This is confirmed using different types of ions, including divalent Ca^2+^ ions that can adsorb not only in the cavity sites available to Rb^+^ ions, but also in the interstitial region between cavities^54^,^57^ (Supplementary Fig. S3). Interpretation of the other two levels (purple and orange arrows) visible in Fig. 2a is less straightforward and involves a combination of H_3_O^+^ adsorption and hydration effects on the surface, which are addressed later in the paper.

In order to objectively interpret the dynamics of these different surface states, it is useful to extract their respective associated timescale. Taking a time profile over a site temporarily occupied by Rb^+^ ions offers a kinetic trace of the Rb^+^ residence time on the site (Fig. 2b). The profile can be analysed using a threshold value carefully selected to be between the heights respectively associated with the main different surface states (with or without adsorbed Rb^+^): each time interval during which the height of the kinetic trace is continuously above the threshold is interpreted as a single residence event with a quantifiable duration. This interpretation requires some implicit assumptions since ions can diffuse laterally on the surface and desorb at the same time (lateral and normal diffusion). The probability for a given residence interval tends to decay exponentially with the duration of the interface. This is to be expected for diffusion processes, and consistent with previous studies of the analysis of the residence time τ of water molecules in the solvation shell of ions^58^,^59^. Our results (inset Fig. 2b) return a characteristic decay timescale τ = 100 ± 7 ms for the residence time of Rb^+^ ions. This is significantly higher than our instrumental limitation of 25 ms, indicating that the AFM measurement can capture some of the intrinsic dynamics of the system.

The analysis of selected profiles such as in Fig. 2b is however limited, because it only provides information over a relatively small number of events, often subjectively selected. The same holds for the choice of threshold value in these conditions. Additionally, the time constant derived offers a poor quality fit of the data (inset Fig. 2b). In order to overcome these limitations, we extended the analysis to each site for the whole duration of the experiment. First, it is crucial to quantify the tip’s lateral drift throughout an experiment such as presented in Fig. 2a. It is convenient to distinguish the drift parallel and perpendicular to the scanning direction. The parallel component is directly visible in as vertical ‘undulations’ of lattice site in Fig. 2a, indicating an maximum drift rate of 15 pm/s. Temporal analysis of the dynamic evolution of each site requires compensation for parallel drift (Fig. 2c,d) which then provides an objective and complete set of data (see also Supplementary Figs S4–S7). However, this analysis relies on the assumption that perpendicular drift is negligible. Quantification of the perpendicular drift rate yield maximum values of 9 pm/s, confirming that the tip remains over the same row of lattice sites throughout the experiment in Fig. 2a (Supplementary Fig. S8). Generally, perpendicular drift can be neglected here.

Comprehensive analysis of the data shows that the resulting residence statistics are best described with an exponential decay comprising three distinct timescales (Fig. 2e). First, a short timescale, τ_0_ = 25 ms coincides with the scanning rate and hence reflects measurement-related noise. A second timescale of τ_1_ = 104 ± 5 ms originates from the residence of Rb^+^ ions, as already observed in Fig. 2b. The last timescale, τ_2_ = 608 ± 30 ms is related to the dynamics of transitions between the two lowest surface levels (purple and orange arrows). Since the transitions are relatively slow (typically several seconds in Fig. 2e), fewer events are counted in the statistics, despite representing a significant part of the measurements. In order to ensure objective and reliable analysis, the statistics were obtained while systematically varying the threshold value over the height range of the bulk of the raw data, between −0.2 nm and 0.1 nm (see supplementary Fig. S5c,d). It was found that the derived timescales only weakly depend on the choice of threshold, indicative of a robust and reliable analysis. The data presented in Fig. 2e were acquired with a −0.1 nm threshold, but the uncertainties given for the timescales reflect their respective statistical variability across the range of thresholds probed rather than the fitting error (see supplementary Table S6).

The slowest timescale is related to the dynamics of H_3_O^+^ and the resulting alteration of the local surface hydration. The hydronium ion H_3_O^+^ can compete with dissolved metal cations for adsorption on the mica substrate^56^,^60^,^61^. However, H_3_O^+^ tends to be located deeper into mica’s ditrigonal cavities than alkali ions due to its smaller size^32^,^55^.

**Figure 3:**
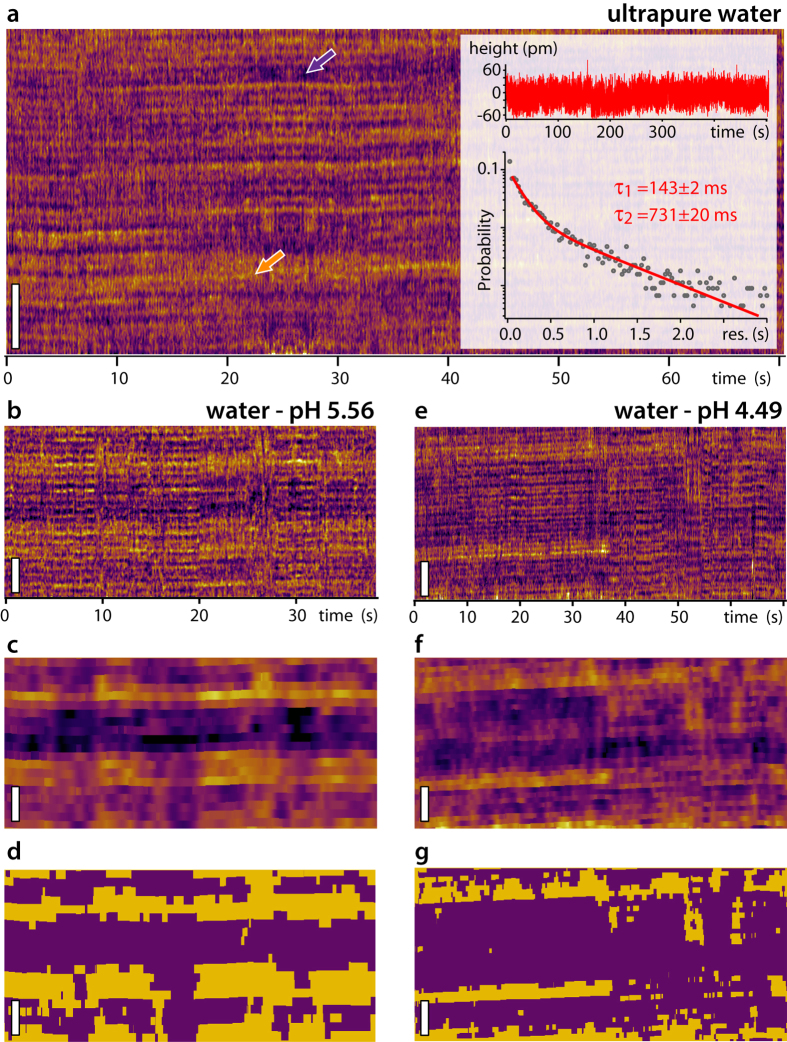
Time evolution of the mica surface in water. Kinetic experiments conducted in pure water (**a**) show mainly two levels (arrows) when compared to Fig. 2a. Height variations are less pronounced than in RbCl solution and analysis of the surface dynamics (inset) reveals slower timescales with a relatively strong dependence on the choice of threshold. The profile shown in the inset is taken after site averaging (see e.g. Fig. 2d), hence the small height variations. More reliable results were obtained for lower threshold values (here −20 pm, see Supplementary Fig. S10). The overall ratio between the two levels visible in (**a**) can be changed by adjusting the pH of the water with HCl (**b–g**), suggesting the higher level to be related to hydration water and the lower level to reflect adsorption of H_3_O^+^, as detected by the AFM tip. For each of the pH value studied, the raw kinetic experiments (**b,e**) are site-averaged (**c,f**) as in Fig. 2d to remove the mica corrugation and imaging noise. The height distribution of the site-averaged data is then binarised automatically (**d,g**) depending on whether the surface height is higher or lower than the average between the surface’s highest and lowest points. The fraction of surface interpreted as covered with H_3_O^+^ (purple in **d** and **g**) changes from 55 ± 3% to 75 ± 2%. (**b**,**e**) were acquired with a same tip. The mica samples have been rinsed with the imaging solution after being cleaved and the presence of K^+^ ions on the surface can be neglected (concentration <10 nM). The scale bar is 3 nm in all experiments.

Alternatively water molecules can locally orient their dipole so as to neutralize mica’s surface charge in the absence of adsorbed ion. Both states always exist when mica is immersed since water can dissociate, but the balance between the two is controlled by the pH of the solution in contact with the surface. Figure 3 shows kinetic experiments identical to that in Fig. 2a, but conducted in ultrapure water (Fig. 3a–d), and ultrapure water acidified with HCl (3e–g).

In water, apparent height variations over the surface are significantly lower than in saline solution, partly due to the particular interplay between the tip and surface hydration structures^7^,^43^,^44^,^45^,^48^,^49^,^62^. Two levels are nonetheless clearly visible (arrows in Fig. 3a) and vary slowly with time. Analysis of the kinetic data (inset Fig. 3a) reveals slower timescales than in saline solutions, sometimes with τ_2_ in excess of seconds. The analysis should be taken with caution because the height variations are close to the imaging noise level, resulting in a relatively strong dependence on the choice of threshold (see supplementary Fig. S10). The existence of longer timescales that for Rb^+^ ions is however a robust result. Repeating the experiment at different pH (Fig. 3b,c) changed the surface ratio occupied by each of the two levels. This indicates that that the lower level is related to adsorbed H_3_O^+^ while the higher to water forming the first surface hydration layer in the absence of ions. The exact hydration structure of the surface cannot be derived from AFM results alone, and a fully quantitative analysis of the pH-induced change of the apparent H_3_O^+^/water surface ratio is questionable given the influence of the AFM tip on the measurement^13^,^49^. Here the problem is mitigated by selecting a pH window where the surface charge of mica changes significantly^63^,^64^, but with limited changes (<10%) to the tip’s negative surface charge^65^,^66^. The results are qualitatively in agreement with available literature^63^,^64^ and the attribution of the different levels is compatible with X-ray reflectivity observations obtained at equilibrium^32^,^55^,^67^,^68^.

In summary, the AFM results suggest that the residence dynamics of Rb^+^ ions is dominated by hydration effects, with H_3_O^+^ and Rb^+^ ions competing for access the hydrated surface’s binding sites. This interpretation is directly visible in Fig. 2a: when following the time evolution of a given site, Rb^+^ ions (yellow arrow) adsorb on sites previously simply hydrated (orange arrow) but not on sites already occupied by H_3_O^+^ ions (purple arrow). A statistical analysis indicates that >92% of the Rb^+^ adsorption events detected by thresholding occur on sites that are simply hydrated, against <8% on sites interpreted as occupied by H_3_O^+^ ions. The dynamics of H_3_O^+^ ions (τ_2_ = 610 ± 30 ms) is considerably slower than that of Rb^+^ ions (τ_1_ = 104 ± 5 ms) on the surface, reflecting the higher affinity of the former for mica^56^.

**Figure 4:**
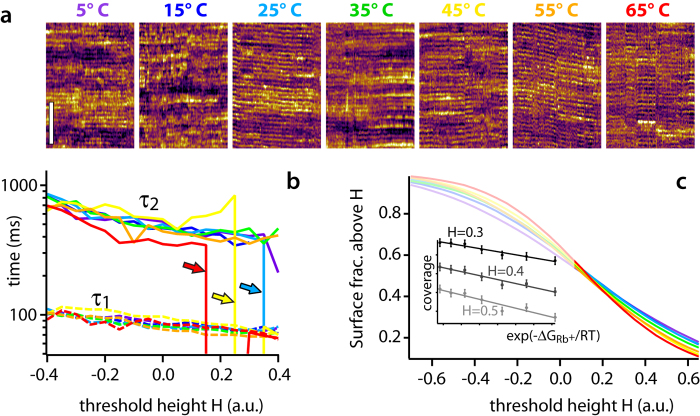
Influence of temperature on the surface kinetics of mica in a solution containing Rb^+^ ions. As the temperature increases, the apparent roughness of mica tends to decrease and the surface becomes smoother, except for possible Rb^+^ adsorption events (**a**). The height of all the kinetic data has been renormalized to allow direct comparison (see also supplementary Figs S11 and S12 for details). The characteristic timescales τ_1_ and τ_2_ calculated from the kinetic traces show little dependence on temperature for a wide range of height thresholds H (**b**). Generally, the timescales tend to decrease with increasing H since higher thresholds emphasize faster adsorbing/desorbing Rb^+^ ions over the slower dynamics of hydration water and H_3_O^+^ in the statistics. For the higher temperatures τ_2_ can decrease rapidly past H~0.1 (arrows), often converging to values close to τ_1_ due to the smoother mica surface that makes distinguishing between the two timescales difficult. The decrease of apparent surface roughness can be quantified by plotting the surface fraction above a given height H for each temperature (**c**). Since the height has been normalized, all the curves in (**c**) intersect in a same point near H~0.1. This ‘isosbestic’ point is not at H = 0 due the adsorbed ions and water molecules at the interface that create an asymmetry in the height distributions (see Fig. S12). The region of the curves >H~0.1 reflect the height of the hydration water and adsorbed Rb^+^ ions in (**a**). As the temperature increases, the surface fraction decreases more rapidly with H, following an Arrhenius-type behaviour (Fig. S12). The scale bar is 5 nm in (**a**) and the kinetic sequences represent 10 s for each temperature (see also Figs S10, 11), and the analysis (**b,c**) represents more than 30 min × site.

In order to further investigate the influence of the environment on the results, we repeated the experiment presented in Fig. 2 while systematically varying the temperature of the solution from 5 °C to 65 °C. The main results are presented in Fig. 4. As the temperature increases, the apparent surface roughness of mica decreases. In the kinetic data (inset Fig. 4a), this is reflected by the mica surface becoming progressively smoother, except for eventual Rb^+^ adsorption. In order to allow for direct comparison between the different temperatures, the height distribution over all the kinetic data had to be renormalized (inset Fig. 4b). The fact that Rb^+^ adsorption remains visible on smoother mica indicates that the apparent roughness is mainly due to interfacial water that becomes progressively more mobile as the temperature increases. This is consistent with previous studies that showed interfacial water to be largely glassy near hydrophilic surfaces^69^,^70^,^71^,^72^. In order to better quantify these observations, it is useful to compare the fraction of surface area above a given height threshold for each temperature (Fig. 4b). The curves all intersect at a same ‘isosbestic’ point that reflects the renormalization procedure: since the absolute height is not known from AFM data, the height distributions have been readjusted and centred around zero by fitting with a reference Gaussian distribution. The shape of the different height distributions is however preserved by the procedure. The height distribution are not fully symmetrical due to the presence of adsorbed ions and water molecules (inset Fig. 4b), and the isosbestic point in Fig. 4b is hence at H~0.1 rather than H = 0. The surface fraction curves (Fig. 4b) show a more rapid decay for higher temperatures, consistent with a smoother mica surface (narrower height distribution). For a given H value in this region (H > 0.1), the change of surface fraction follows an Arrhenius behaviour with temperatures (Fig. S12), consistent with a reduction in glassiness of the interfacial water.

The impact of temperature on the characteristic timescales of the surface appears minimal (Fig. 4c). The timescales are marginally shorter for τ_2_ at higher temperatures, with the fitting procedure tending to make both timescales converge to τ_1_ past a certain threshold (arrows in Fig. 4b). This is consistent with the mica surface becoming progressively smoother, making τ_2_ difficult to extract, especially for higher threshold values.

Despite limited results regarding the evolution of the Rb^+^ residence time with temperature, the experiment indicates that the effect is the most important on the hydration water. Since these water molecules modulate the state of the surface in the competitive Rb^+^/H_3_O^+^ adsorption, temperature might act indirectly on ion adsorption. Once an ion is adsorbed to the mica surface, the associated residence time shows little dependence on temperature within the range studied here, but the behavior of interfacial water may modulate the frequency of the adsorption events. The data available here is however not sufficient to offer a definitive answer to this question and further studies are needed to fully address this point.

## Discussion

The timescales identified in this study are relatively slow and suggest that the protrusions imaged by AFM represent single adsorbed ions rather than ionic densities. Considering a typical bulk diffusion value of *D*_*b*_ ~ 10^−9^ m^2^ s^−1^ for monovalent metal cations such as Rb^+^ in water^73^, the diffusion time between adjacent sites on mica would be in the order of tens of μs. This simple consideration ignores any specifics about the interface, in particular the role of surface hydration known to play an important role^13^, and the existence of specific ion binding sites on mica. At the present time, there exists no model able to take these interfacial effects fully into account, but a first approximation can be derived by adapting developments from the Transition State Theory. If we consider only ion adsorption as the main mechanism able to slow down the diffusion of ions at the interface, the interfacial diffusion coefficient Di can be expressed as a function of D_*b*_^74^:


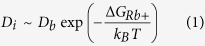


where *ΔG*_Rb+_ is the adsorption free energy of Rb^+^ ions to the surface of mica in water (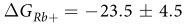 *kJ mol*^-1^)^13^,^60^. This approach is an oversimplification since it neglects any specific interaction or directional effects at the interface, but it provides an order of magnitude for the expected timescales. Depending on the temperature, the Arrhenius factor varies between 4 × 10^−5^ (5 °C) and 2.3 × 10^−4^ (65 °C), giving interfacial diffusion coefficients values in the order of *D*_*i*_ ~ 10^−14^–10^−13^ m^2^ s^−1^. This corrected diffusion yields timescales in the order of tens of milliseconds and up to seconds for an ion to move between adjacent sites, consistent with the AFM observations.

One of the key findings of the present study is the importance of hydration water – of both the mica surface and the adsorbed ions– in modulating the apparent interfacial dynamics. The life time of a single hydrogen bond (typically 1–2 ps^75^) is extremely fast compared to the timescale identified here, but translational motion can be substantially slower due to the need for a collective rearrangement of the hydrogen bond network. Water’s self diffusion coefficient in bulk and at room temperature, 2.3 × 10^−9 ^m^2^/s^76^, is comparable to that of alkali ions in bulk of aqueous solutions^73^. At the interface with mica, these dynamics can be expected to slow down by several orders of magnitude due to water-mica interactions. Applying a reasoning similar to that derived above for Rb^+^ ions and considering the hydration free energy of mica^13^,^77^, the expected reduction of translational mobility for water molecules at the interface is even more pronounced than for adsorbed ions. The mobility of the water molecules hydrating the adsorbed ions depends strongly on the ion’s size and charge with average residence time in the primary hydration shell varying from picoseconds to years^78^. At solid-liquid interfaces, ion diffusion is hindered both by the presence of a solid, and by the specific hydration landscape encountered. Surface hydration water interacts with the hydration shell of ions, which can result in substantial stabilization effects. In previous work, we showed water-induced attractive correlation effects between adsorbed Rb^+^ ions at the surface of mica^13^. Here we show that Rb^+^ ions can remain for τ_1_~100 ms at a same adsorption site of mica’s surface. Although experimental limitations do not allow us to capture possible faster dynamics, the fact that the AFM tip only requires few microseconds to generate a pixel over a given location, the high reproducibility of the results as well as simple theoretical considerations suggest that that AFM does indeed image single adsorbed ions and not highly mobile ‘ionic densities’ on the surface^53^.

In any case, the AFM results unambiguously show that the residence of Rb^+^ ions on single atomic sites are characterized by a ~100 ms timescale, but also depend on the hydration state of the surface which varies on a slower τ_2_ > 600 ms timescale. The shortest timescale τ_1_ was reproducibly observed on different AFMs, and using different types of AFM tips (Figs S13 and S14). The longer timescale τ_2_, ascribed here to the residence of H_3_O^+^ ions on the surface, effectively modulates the availability of binding sites for Rb^+^ ions. The difference between the two timescales is consistent with the fact that the equilibrium constant for adsorption for individual ions on mica is about ten times larger for H_3_O^+^ than for Rb^+^ at room temperature^56^. Changing the system’s temperature does not appear to affect τ_1_, but rather the apparent roughness of the interface (Fig. 4), with the hydration water exhibiting an Arrhenius-type behaviour (Fig. S12b). At lower temperatures, the interface appears rougher due to glassy water, forming layers that can be easily imaged by the AFM tip. As the temperature increases, the water molecules become more mobile and the surface appears smoother. The associated τ_2_ timescale partially follows this trend by decreasing with increasing temperature, but no firm conclusion can be reached due to the experimental error of our measurements. We explain the glassy behaviour of water by the collective effect of molecules composing the hydration landscape of mica. Here the strong affinity of water for the mica surface may contribute to this effect by stabilizing interfacial water molecules. Ice-like water has indeed been reported at the surface of mica at room temperature^79^ although with the surface not immersed in liquid water. Recent results have demonstrated that molecular group effects can dramatically stabilise hydrogen-bond networks at interfaces^80^. Here, this view could also explain the fact that, within error, τ_1_ remains unaffected by temperature; the hydration structure immediately surrounding adsorbed ions is likely to be less sensitive to temperature variations given its localized nature and the fact that it involves fewer molecules. Hydration water surrounding ions rarely behaves as bulk water with respect to temperature^81^,^82^. The present results suggest that both the local affinity of the water for the surface and the ability to develop a well-defined hydration structures^41^,^43^ play an important role in controlling interfacial dynamics, but both effects are not affected similarly by temperature, at least within the range probed here. In other words, the interfacial dynamics of Rb^+^ ions seem to be dominated by diffusion effects rather than classical adsorption/desorption models, something that we attribute to the unusual properties of interfacial water.

The measurement method can in principle also affect the results since AFM is an intrinsically perturbative technique^13^,^41^,^48^. Using AFM alone, it is not possible to entirely rule out the possibility that the derived timescales depend on the mode of measurement (i.e. that the cantilever tip modulates τ_1_ and τ_2_). The tip can induce a local increase in the ionic concentration of the solution as well as charge regulation effects due to its own surface charge^66^. Possible confinement of ions between the tip and the surface could in principle affect the observed dynamics^83^,^84^. However, the tips used for the measurement are relatively sharp, judging by the resolution achieved, and the results are therefore likely to be dominated by the sample’s interface^85^. On similar system at equilibrium, AFM and molecular dynamics simulations are often in good agreement^12^,^13^,^86^,^87^,^88^,^89^,^90^. Additionally, after scanning any given site, the tip moves to a distance larger than the range of electrostatic or hydration interactions in water (>10 nm) before revisiting it while scanning. This leaves several milliseconds for any given point of the interface and adsorbed ions to relax or diffuse unimpeded, before a new pass of the tip at the same site. Finally, the important difference between the derived timescales and that of the measuring tip suggest τ_1_ and τ_2_ to represent the intrinsic dynamics of the system. These results have profound implications for interfacial processes involving electrolyte solutions. The existence of stable ionic structures are bound to impact on charge transfer at interfaces, for example in electrochemical measurements^91^, in the development of batteries^92^,^93^,^94^ or in bioenergetics^95^. In soft matter and biology, these results could have even more important implications since adsorbed ions also influence the mechanical properties of materials, for example the case of biological membranes^38^,^39^,^96^. The hydration landscape of mica is very similar to that of ordered lipid domains^46^,^97^,^98^ and long-lived ionic structures are likely to influence their evolution as well as the overall shape^14^,^39^ and properties^38^,^96^,^99^,^100^ of the membrane.

In conclusion, we have used high-resolution AFM with 25 ms resolution to follow the adsorption/desorption dynamics of single alkali metal ions at the surface of mica in aqueous solution. The results show remarkably long residence for Rb^+^ that stay more than 100 ms on the surface. The process is mediated by the interfacial water and competing H_3_O^+^ ions that evolve on a slower (>600 ms) timescale. The ability of AFM to probe solid-liquid interfaces locally, with sub-nanometre spatial resolution and millisecond temporal resolution opens many possibilities for investigating molecules dynamics at interfaces, in particular for system currently beyond the reach of computer simulations. These results reported here have important implications for the understanding and modelling of interfacial processes, and are likely to extend far beyond the model system studied in this paper.

## Materials and Methods

All atomic force microscopy results were conducted using a Cypher ES (Asylum Research, Oxford Instruments, Santa Barbara, CA, USA) except for the results presented in Fig. S10 that were obtained on a Multimode IIIA (Digital Instruments, now Brucker, Santa Barbara, CA, USA). All measurements were performed with the cantilever/tip and the sample fully immersed in solution. The measurements with the Cypher were thermally stabilized (±0.1 °C) and the tip oscillation was driven photo-thermally for greater stability. We used two different types of cantilevers: Arrow UHF-AUD (Nanoworld, Neuchatel, Switzerland) with a spring constant of 2–3 N/m and RC800PSA (Olympus, Tokyo, Japan) with a spring constant of 0.6–0.8 N/m. No significant differences were noticed between the two types of cantilevers, but the smaller Arrow levers allowed for higher frequency in liquid (~400 kHz) which is beneficial to fast measurements. The AFM was operated in amplitude modulation with working amplitudes *A* between 0.5 nm and 1.5 nm and a setpoint ratio *A*/*A*_*0*_ > 0.7, where *A*_*0*_ is the free vibration amplitude of the tip away from the interface. In these conditions the phase lag φ between the driving vibration and that of the tip is sensitive to the behaviour of the liquid expelled by the vibrating tip and its affinity for the surface^7^,^41^,^43^,^44^, and the resolution is enhanced by short-range solvation forces^12^,^45^,^47^,^101^,^102^,^103^. The imaging working amplitude was however kept larger than ~0.5 nm so as not to sweep aside weakly adsorbed ions while imaging^12^,^13^.

All the reagents (purity > 99%) were purchased from Sigma-Aldrich (Dorset, UK) and used without further purification. Grade I mica was obtained from SPI supplies (West Chester, PA, USA) and freshly cleaved with adhesive tape before each experiment. It was then rinsed extensively with the experimental solution before being covered by a drop (150 μL) of the same solution used for the experiment. The solutions made with ultrapure water (18.2 MΩ, Merck-Millipore, Watford, UK).

## Additional Information

**How to cite this article**: Ricci, M. *et al*. Direct observation of the dynamics of single metal ions at the interface with solids in aqueous solutions. *Sci. Rep.* **7**, 43234; doi: 10.1038/srep43234 (2017).

**Publisher's note:** Springer Nature remains neutral with regard to jurisdictional claims in published maps and institutional affiliations.

## Supplementary Material

Supplementary Information
