# Supplementary material for: Direct observation of the dynamics of single metal ions at the interface with solids in aqueous solutions
Source: Sci Rep. 2017 Feb 23;7:43234. doi: 10.1038/srep43234 (PMC5322364; doi:10.1038/srep43234)
Supplement: Supplementary Information [file srep43234-s1.pdf]

Supplementary Information

Direct observation of the dynamics of single metal ions at the interface with solids in aqueous solutions

Maria Ricci[1], *William Trewby*[2], *Clodomiro Cafolla*[2], *Kislon Voïtchovsky*[2]*

1 University of Cambridge, Cavendish Laboratory, Cambridge CB3 0HE, UK

2 Department of Physics, Durham University, Durham DH1 3LE, UK

*correspondence: kislon.voitchovsky@durham.ac.uk

Content:

Supplementary figures S1-S14 with extended captions

Supplementary references

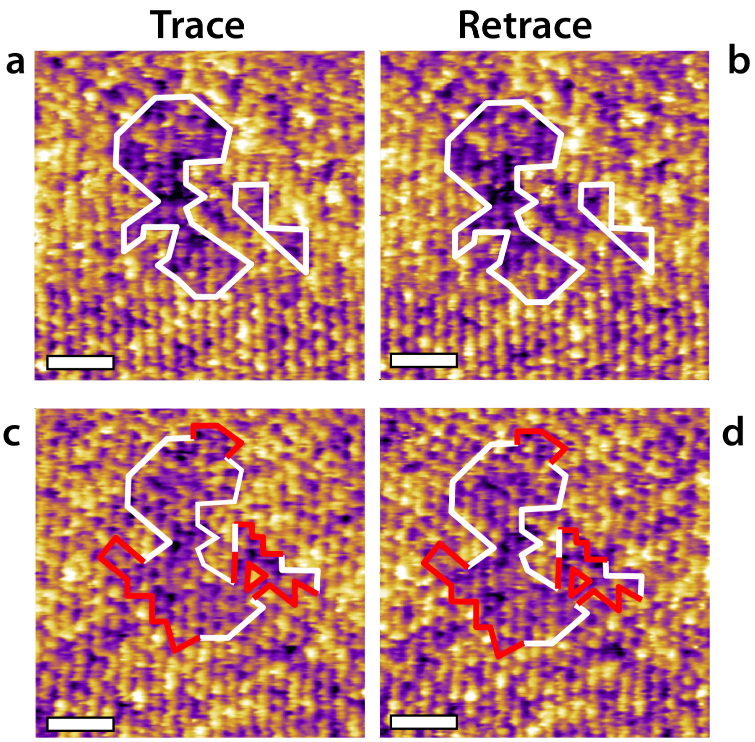

Figure S1: Topographic images of Rb^+ ions adsorbed at the surface of mica in aqueous solution. The Rb^+ ions appear yellow while the mica surface is purple. (a-b) and (c-d) were acquired consecutively at the same location. As for Fig. 1, (a) and (b), respectively (c) and (d) were acquired with the tip scanning from right to left (trace) and left to right (retract) as it moves back. The average time between a same location in (a) and (b), respectively (c) and (d) is 45 ms. A gap in the Rb^+ layer is highlighted with a white line in (a-b). Changes in the ion's distribution can be seen 23 s later, in (c-d). The changes are highlighted in red. The scale bar is 3 nm and the color scale represents 0.3 nm height variation.

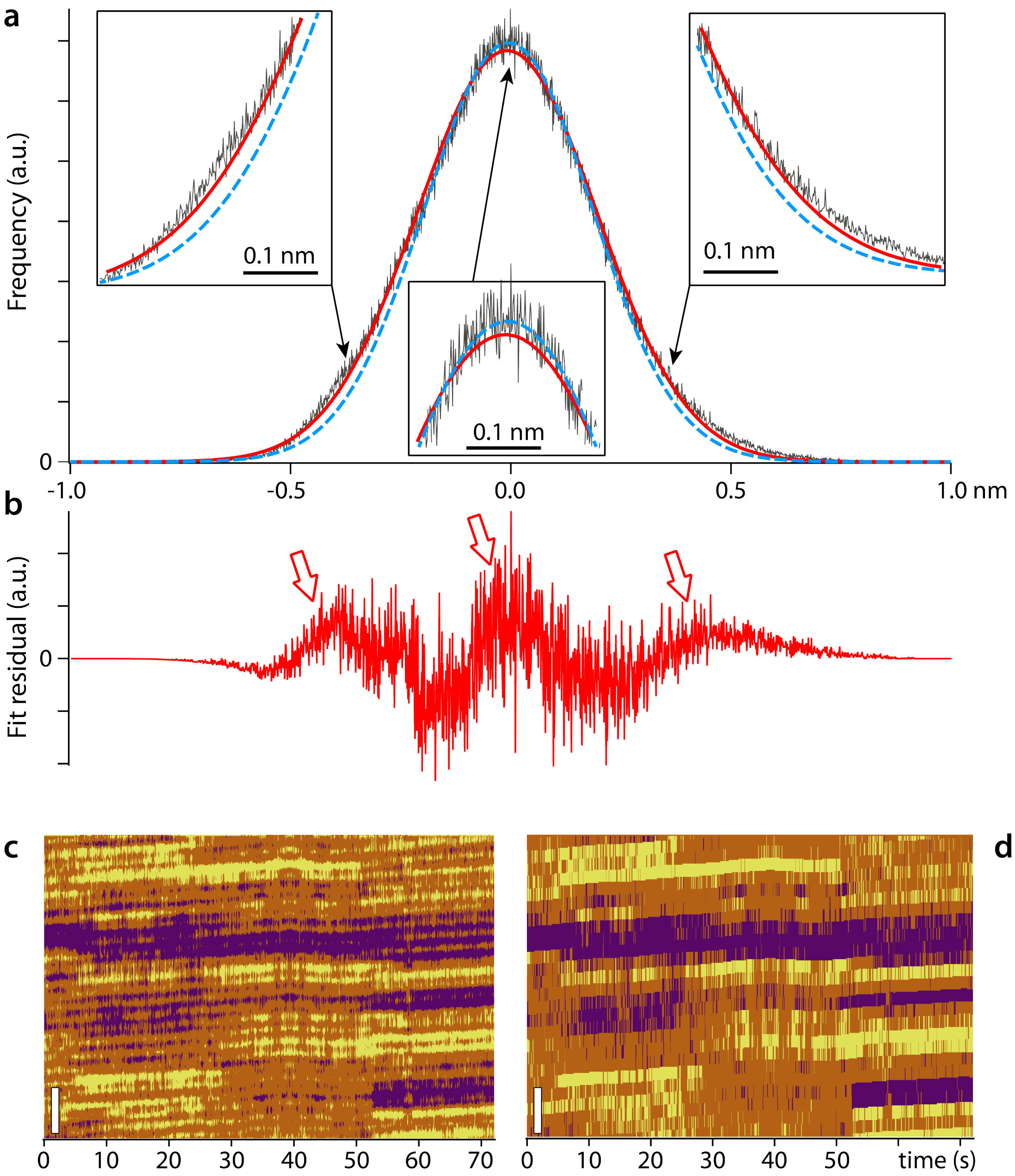

Figure S2: Histogram analysis of the height distribution of the kinetic data presented in Fig. 2. Given the corrugation of the mica surface and the imaging process which is dominated by hydration interactions between the tip and the interface[1-5], it is not possible to distinguish clearly 3 levels in the height histogram (a). However, fitting of the height distribution with a Gaussian distribution (red curve) is not satisfactory and the fitting differ significantly is only the central part of the distribution is selected (blue dashed curve). The residuals (b) of a global fit (with red curve) show three distinct maxima (arrows in b) that can be ascribed to the different levels in Figure 2. Based on the levels identified in (b) the kinetic data can be leveled in three levels. When this analysis is conducted on the raw data (c), the result is qualitatively very similar to un-processed data (Fig. 2a) hence validating the approach. The same analysis conducted on site-averaged data (d) does not show significant differences. The scale bar is 3 nm in (c-d).

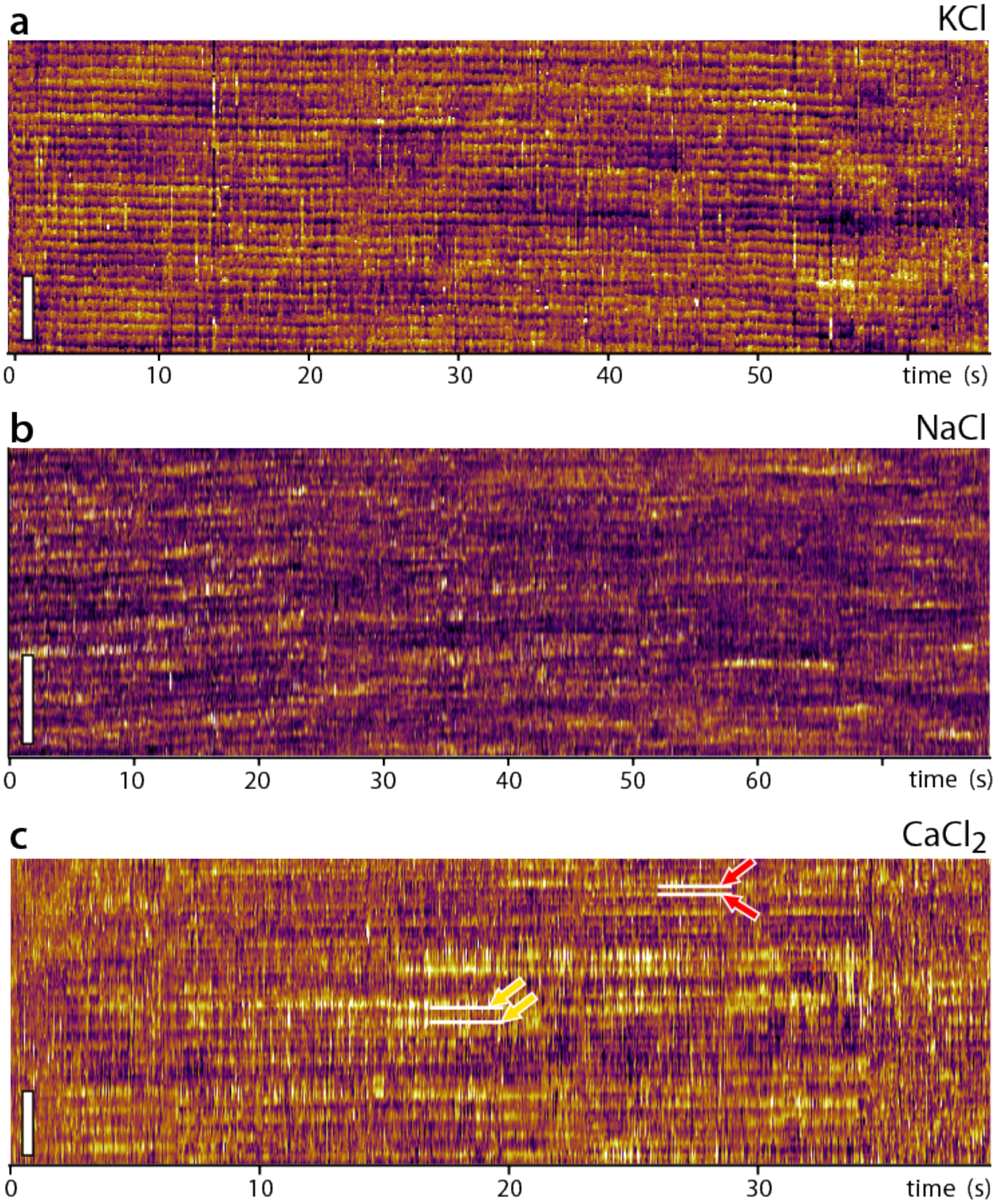

Figure S3: Representative kinetic experiments conducted in KCl, NaCl and $CaCl_2$. Measurements in KCl show remarkably stable imaging conditions, as could be expected from its similarity with Rb^+ in terms of hydration[5]. The distinction between different surface levels is not as clear as in RbCl but slow dynamical changes (>5 s) can be seen. Results in NaCl and $CaCl_2$ are considerably noisier and an interpretation of the dynamics is less straightforward. This is to be expected considering the multiple hydration states of the ions[6,7]. The measurement in $CaCl_2$, show that Ca^{2+} ions can adsorb either in the main sites (yellow arrows) or in interstitial sites (red arrows), between mica's ditrigonal cavities, a result consistent with the literature[6-8]. Since interstitial adsorption was not observed for Rb^+ and K^+, this result, confirms that the brightest level visible in the measurements are indeed due to the adsorbed metal ions. However, given the presence of multiple hydration states, no time analysis was conducted for the ions shown here.

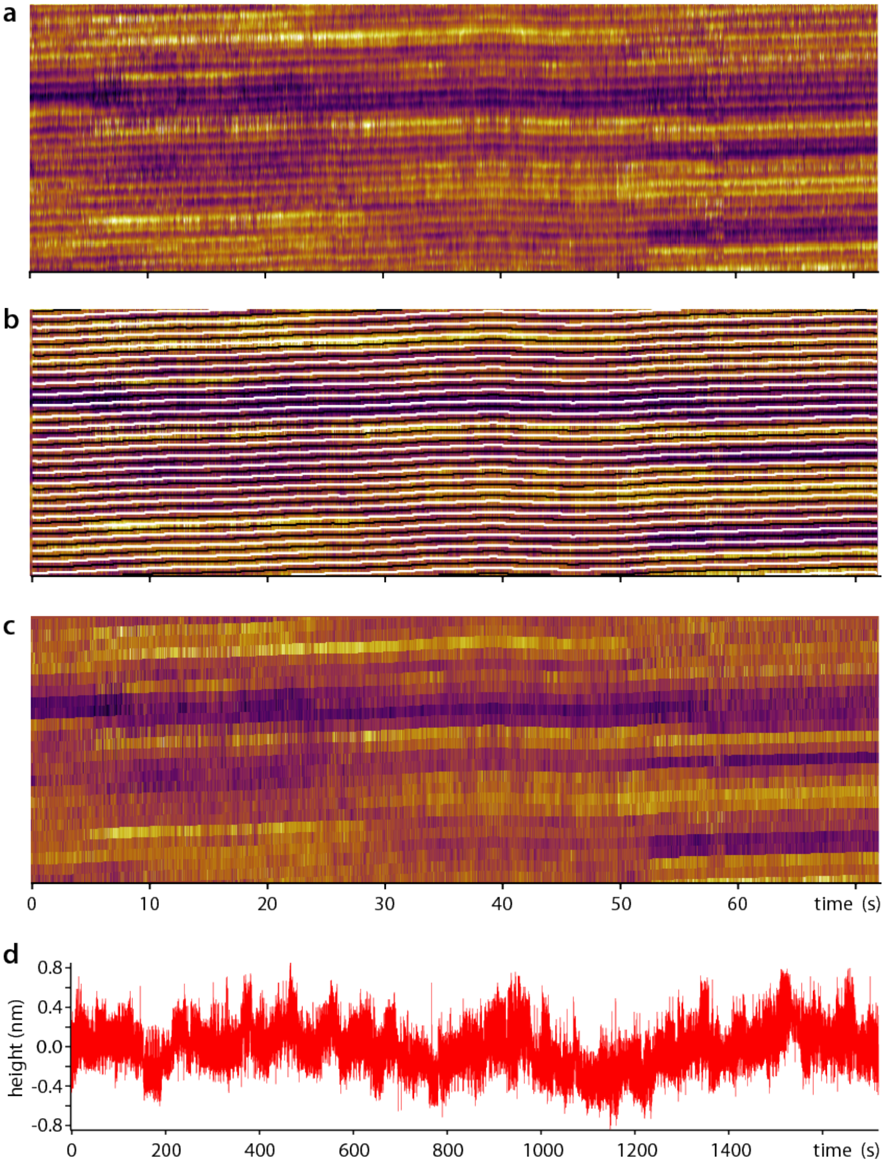

Figure S4: Methodology for analyzing the kinetic data collected by AFM. A typical series (Fig. 2a) is obtained by stitching together consecutive scan lines acquired over a same site along the mica's (100) direction. Each line is 'flattened' by subtracting any tilt and occasional noise spikes are removed (a). The first step of the analysis is to track each site separately throughout the experiment, taking into account any possible drift. A line-by-line Fourier analysis allows clear identification of the underlying mica lattice for each scanned line (b). The routine used for this analysis distinguishes sites (white lines) from inter-site locations (black lines). For each scan line, the region associated with a given site (i.e. between two adjacent black lines) is then averaged to reduce noise (c). The evolution of each site is then obtained by following the average height value along the white lattice lines visible in (c). Stitching end to end the evolution of each site for the whole series provide a singe kinetic trace containing all the information (d). Here the kinetic trace shown in (d) compiles the information of two series conducted during a same experiment resulting in about 30 min × site information, but retaining the 25 ms time resolution. This approach allows full use of the AFM data so as to provide meaningful statistics. The kinetic trace is then analyzed using a standard thresholding approach, presented hereafter in figure S5

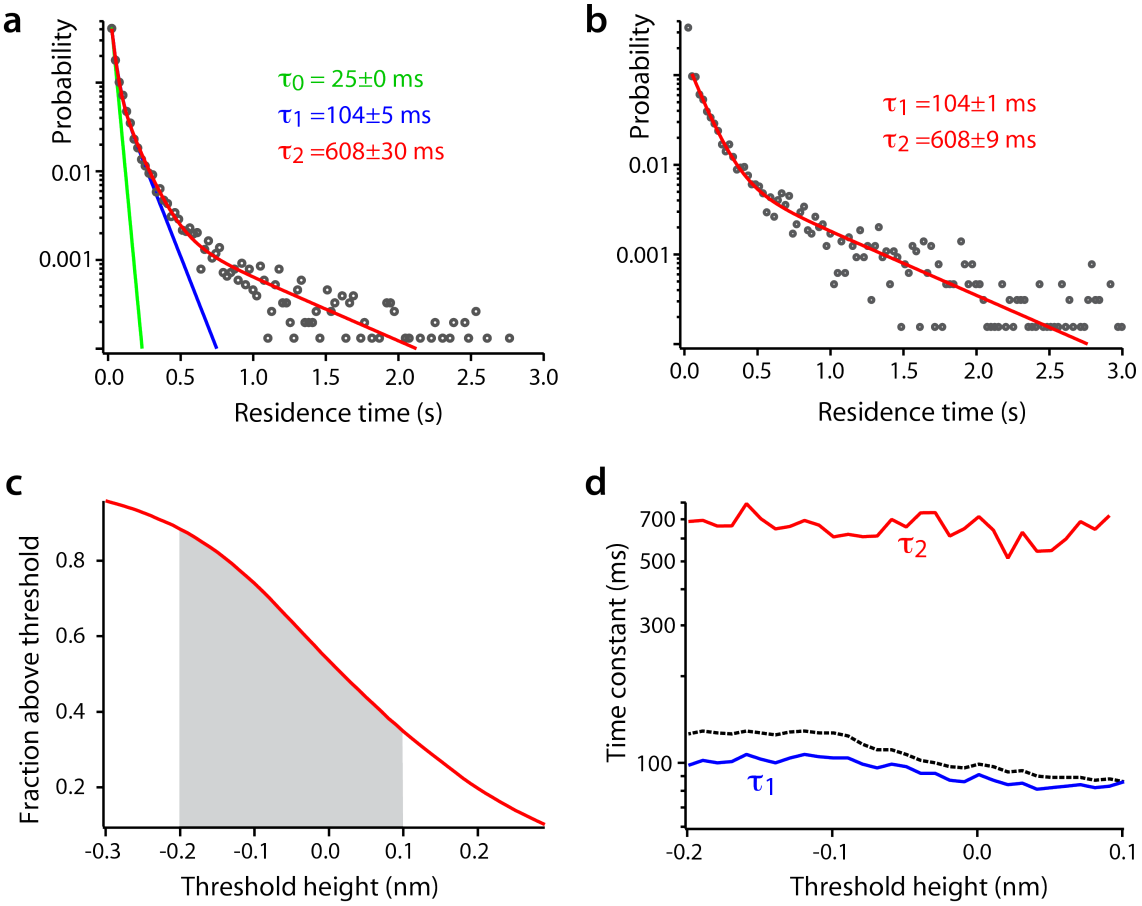

Figure S5: Analysis of the kinetic information extracted in figure 2. Using a given threshold height, statistics is derived on the duration of each time interval during which the height of the kinetic trace is continuously above the threshold (a-b). For the analysis, the distribution of these 'ion residence intervals' can be fitted with a triple exponential decay (a), here for a threshold value of -0.1 nm. The first timescale is invariably τ_0~25 ms (here imposed) and reflects fluctuations between two consecutive scan lines. It is therefore an instrument-related timescale and does not contain information about the system. The second τ_1 and third τ_2 timescales reflect genuine information about the system, interpreted as related respectively to Rb^+ dynamics and surface hydration dynamics. Fitting with a triple exponential is however prone to convergence issues and the same results can be derived via a more robust approach (b). The kinetic trace is corrected so as to exclude any height fluctuations shorter or equal to 25 ms (effectively the first point on the curve) which are replaced by an interpolation between the previous and successive points. The resulting statistics (b) can be reliably fitted with a double exponential, yielding identical values as in (a) within error. The effect of the selected threshold height was systematically investigated with (c) showing the fraction of the surface above the threshold value. The grey interval highlighted in (c) indicates the threshold values for which τ_1 and τ_2 have been calculated in (d). Results show little dependence on the threshold value (d), reflecting the reliability of the findings. The dotted black line in (d) is the time constant derived from a single exponential fit which provides a value close to τ_1. Generally, higher times constants are observed for lower threshold, suggesting that the slower processes linked to the lower surface levels (Fig. 2a). Consistently, for threshold values above ~0.1, τ_2 tends to converge to the value of τ_1, indicating that the higher level is dominated by τ_1. The fitting parameters for (a) are summarized in Table S6.

	Pre-factor A	SD on A (fitting)	Timescale τ [ms]	SD on τ (fitting) [ms]	'Real' error on τ (statistics) [ms]
1st exp.	0.8514	0.0021	25.00	0.00	0
2st exp.	0.1244	0.0008	103.69	0.99	5
3st exp.	0.0033	0.0002	608.40	29.01	30

Note: SD is for standard deviation. The parameters with a zero error are constrained.

Table S6: Fitting parameters obtained from the fitting presented in Fig. 2e and Fig. S5a with a threshold value of -0.1 nm. The equation used for the fitting is an exponential with 3 timescales:

$$P(t) = A_1 e^{-\frac{t}{\tau_0}} + A_2 e^{-\frac{t}{\tau_1}} + A_3 e^{-\frac{t}{\tau_2}}$$

where $P(t)$ is the probability to observe a certain time interval of duration t and A_0, A_1, and A_2 are the 3 pre-factors associated respectively with the 3 timescales τ_0, τ_1, and τ_2. The use of a triple exponential function is often subject to convergence problems due to the large number of parameters, unless some constrained are applied. The statistical analysis was hence conducted primarily with the method described in Fig. S5a-b, using a double exponential fitting that excludes the initial point associated with the 25 ms timescale (see Fig. S5 and caption).
The derived values of the pre-factors A_1 and A_2 typically show a ratio of ~10-50. Their evolution across the probed threshold interval of is shown in Fig. S7. For the larger threshold value, less residence intervals are effectively included in the statistics and the fitting procedure tends to aggregate the data associated with τ_0 and τ_1. In other words, A_0 appears to increase at the cost of A_1. Generally, given the convergence issues associated to the triple exponential fitting, and a larger sensitivity of the pre-factors to the initial fitting conditions, the emphasis was placed on the timescales in the statistical analysis. The timescales were found to be considerably more robust and less dependent on the fitting conditions.
The table also gives the non-rounded timescales values with their standard deviation from fitting. The 'real' error was calculated from statistical variability of the timescales across the interval of thresholds probed. It is represents half a standard deviation. Taking half a standard deviation is justified by the broad width of the interval; it is equivalent to about a full standard deviation for an interval of ±0.1 nm around the threshold selected, where the timescales are the less variable.

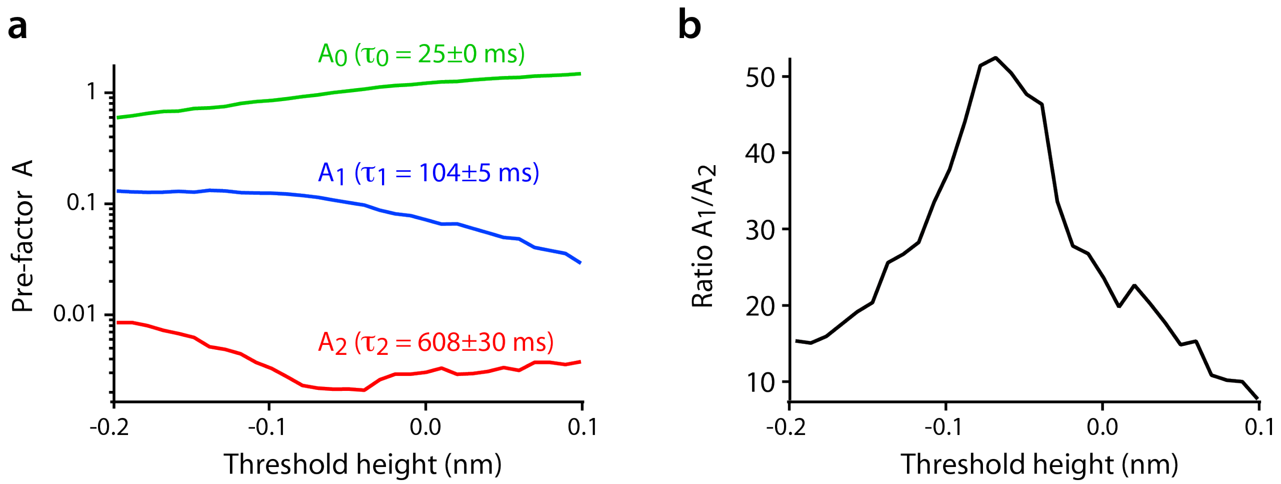

Figure S7: Evolution of the pre-factors A_0, A_1, and A_2 are the 3 associated respectively with the timescales τ_0, τ_1, and τ_2 as a function of the threshold (a). The ratio between A_1, and A_2 is shown in (b), with a typical value of 10-50. The order of magnitude difference between A_1, and A_2 reflects the larger statistical number of short residence intervals τ_1 over τ_2. This should however be considered keeping in mind that the total duration of the event considered (described by intervals τ_1 or τ_2) is comparable since τ_2 is about an order of magnitude larger than τ_1.
The analysis of the pre-factors is less reliable than that of the timescale due to the fact that they are more prone to diverge in the fitting process.

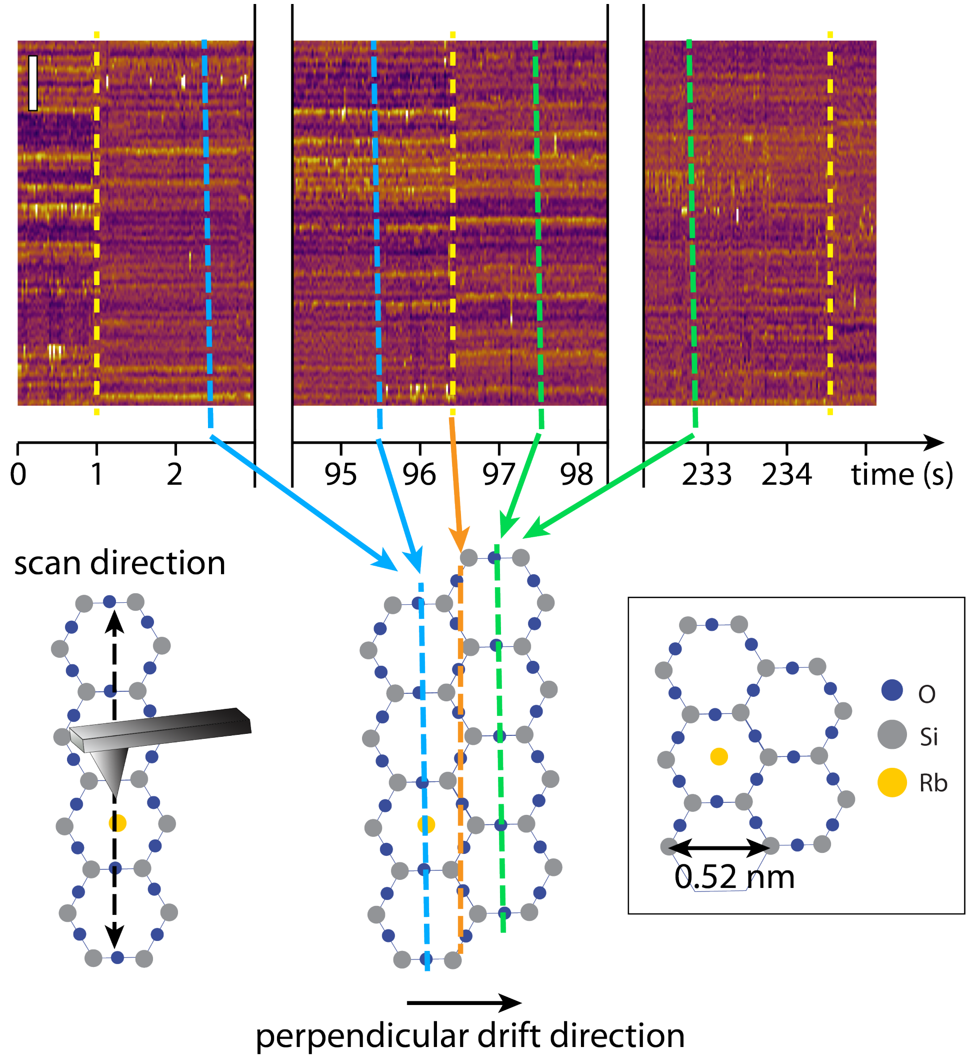

Figure S8: Evaluation of the tip drift rate perpendicular to the scanning direction. The yellow dashed lines show transition points where the scanning tip drifts from a row of lattice sites (dashed blue lines) to the adjacent row (green dashed lines). Due to the hexagonal mica lattice, the two adjacent rows are offset by half the lattice periodicity (~ 0.26 nm). The transition (yellow dashed lines) therefore translates as a sudden vertical shift of ~ 0.26 nm in the imaged profiles. After the transition, new sites appear occupied while previously occupied sites appear suddenly vacant, confirming that tip images a different lattice row. Assuming a drift distance of 0.52 nm between two subsequent transitions and knowing the associate time lapse $T_{perp.}$, it is possible to derive an upper estimate for the drift rate $D_{perp.}$ perpendicular to the scan direction. We found $D_{perp.}$ ~ 9 pm/s ($T_{perp.}$ = 117±20 s). The value of $T_{perp.}$ varied between measurements suggesting that the drift is not continuous and unidirectional, but oscillate around an 'equilibrium' position similarly to the parallel drift. We note that this remarkable stability is, on our opinion, due to the excellent temperature control (better than 0.1 C) and the design of the AFM. These experiments allow us to fully exclude drift as a potential influence on our results since most sequences are shorter than $T_{perp.}$. Drift parallel to the tip scan direction is also unimportant because the analysis procedure tracks the evolution of each site with time (Fig. S4), and hence compensates for parallel drift. The data presented in this figure comes from a single continuous sequence acquired at 40 Hz in 1 mM RbCl. The scale bar is 3 nm.

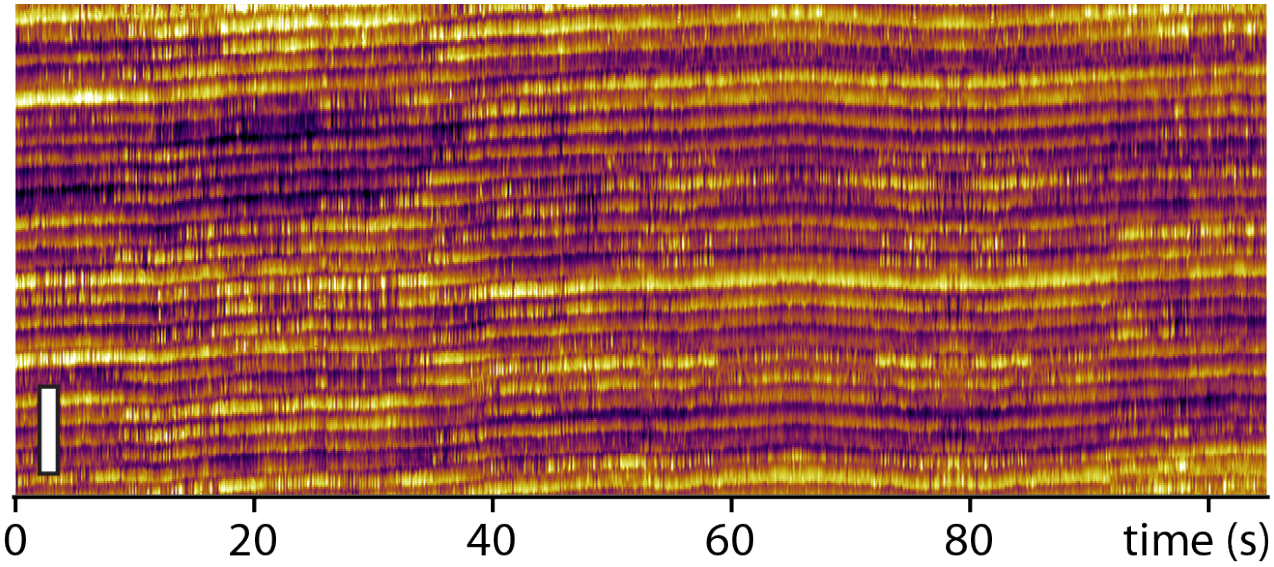

Figure S9: Additional high-resolution kinetic data acquired in 1mM RbCl. The measurement conditions are identical to those used for the results presented in Fig. 2a. The AFM tip is slightly different than in Fig. 2a, providing a different imaging contrast. Three levels are still visible and statistical analysis of the different timescales is consistent with Fig. 2. All the data (this figure and Fig. 2a) have been combined in Fig. 2e. The scale bar is 3 nm.

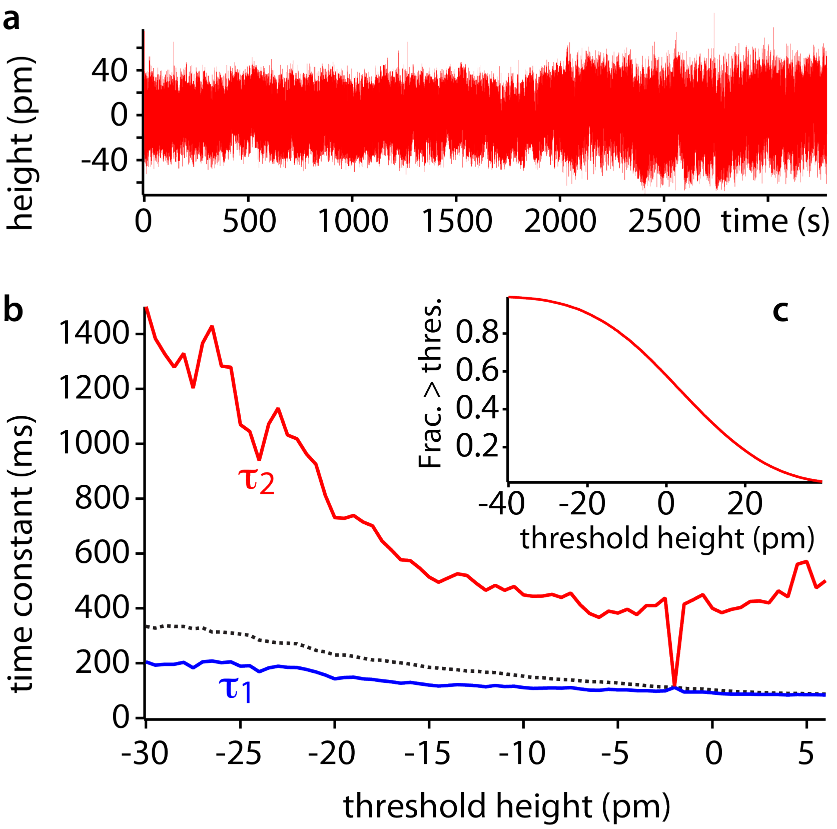

Figure S10: Analysis of the kinetic data obtained in ultrapure water. The height variations along the derived kinetic trace (a) are small compared to height variations in the presence of metal ions. Analysis of the trace yields timescales strongly depending on the threshold value (b), especially when compared to Fig. S4d. As for Fig. S4, the analysis with a single exponential (black dotted) curve is also provided. Generally, the existence of two levels and the fact that height variations are close to the measurement noise levels pushes this type of analysis to its limit, especially at higher threshold values where more data is excluded from the analysis and noise has more impact. A transition is visible around -20 pm, above which the results are mostly dominated by noise. This is confirmed by the identical values derived for τ_1 and τ_2 at -3 pm. The timescales derived at threshold values < 20 pm are significantly larger than for RbCl-containing solutions. The inset (c) shows the fraction of the surface above the threshold as a function of the threshold value.

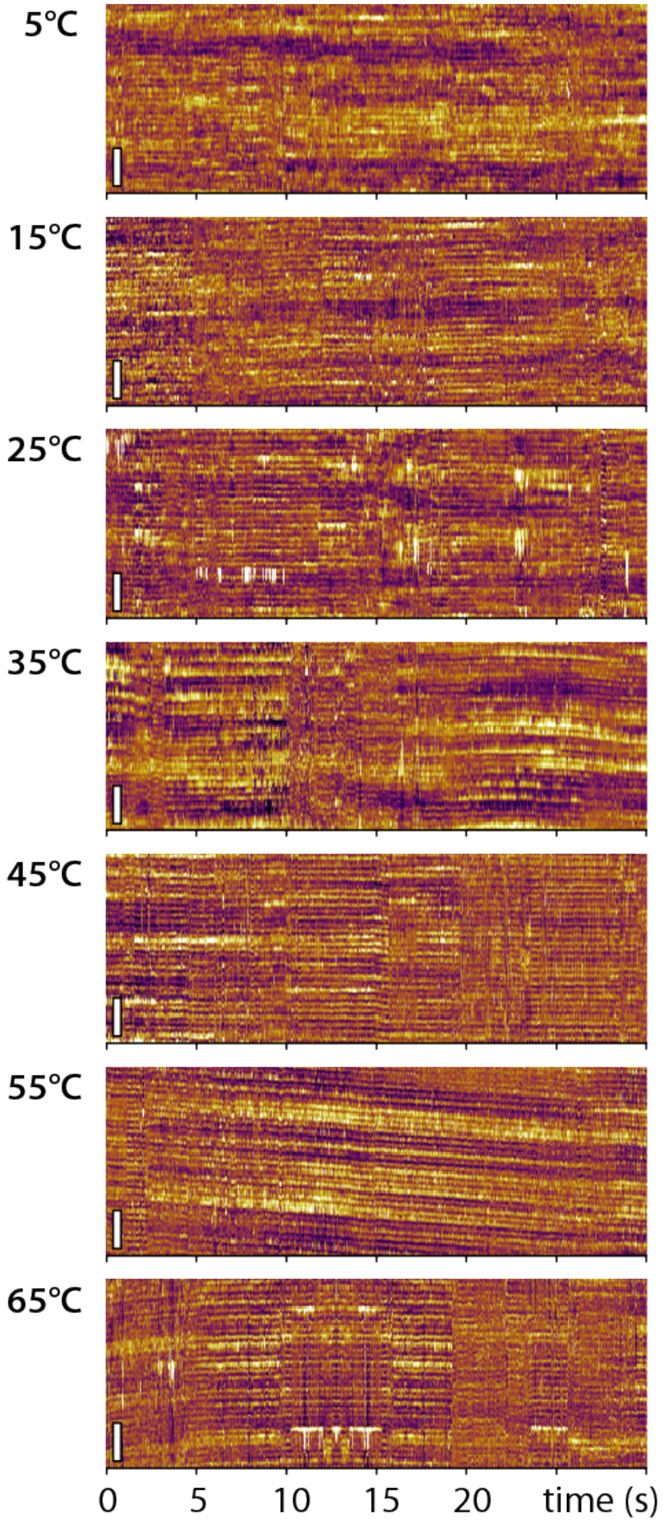

Figure S11: Example of kinetic measurements acquired at different temperatures. In each case, 30 s of continuous recording are shown. Occasional jumps in the resolution are visible, but they do not significantly affect this analysis since their frequency is much lower than that of ionic adsorption/desorption. All the data was acquired during a single set of experiment and with a same tip. Detailed analysis of the measurements is presented in Fig. S7.

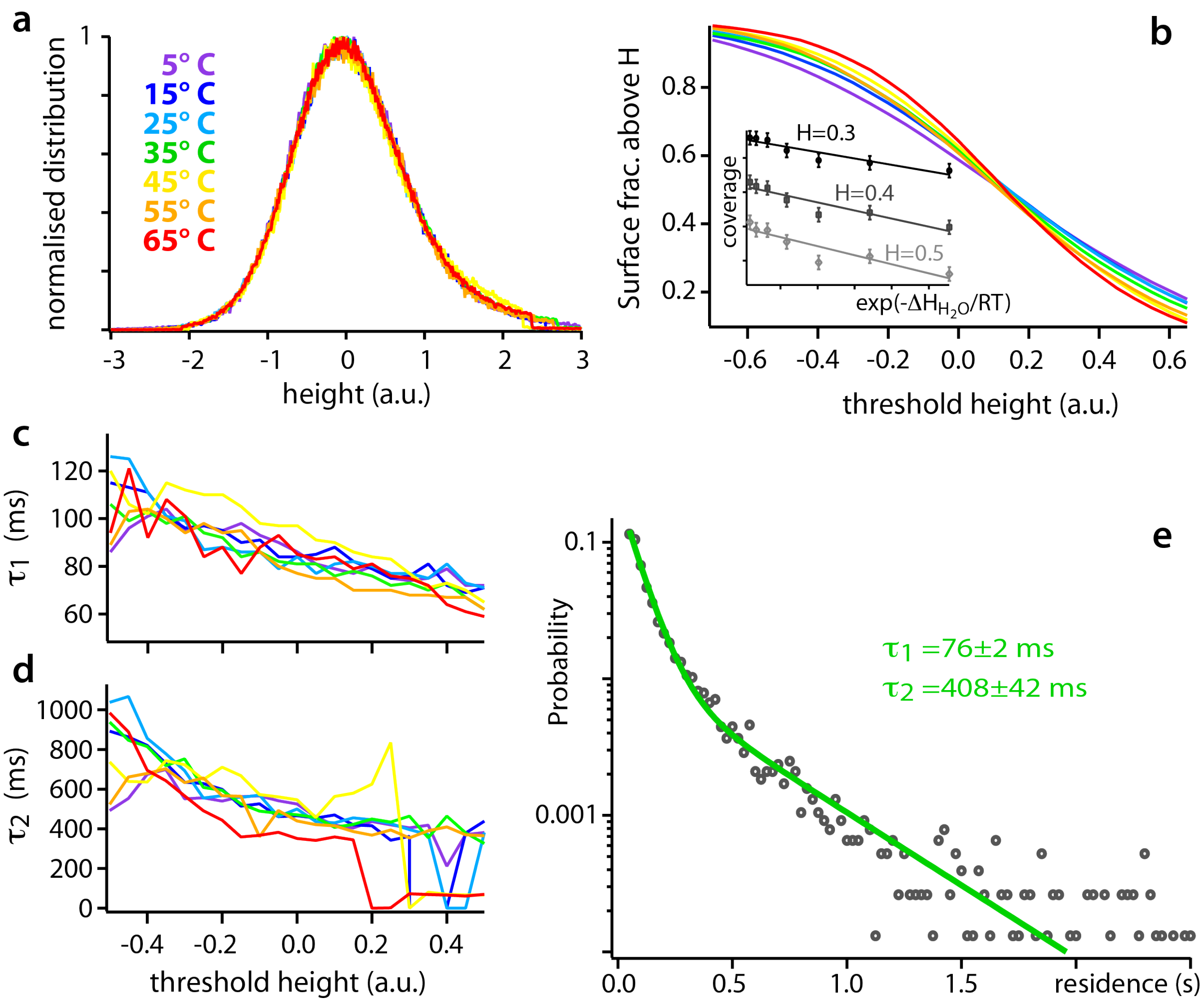

Figure S12: Effect of temperature on the kinetics of the interface in the presence of RbCl. In order to allow meaningful and direct comparison between thresholding over all the sets of data, the height distribution of each set of kinetic data is normalized to a reference Gaussian curve (a). This necessary since each scan line has been corrected for tilt, thereby affecting its absolute height. The shape of the height distribution is however slightly different in each case, with a large tail on the right for colder temperatures. This is visible when plotting, for each temperature, the surface fraction above a given threshold value (b). A more abrupt decrease of the surface fraction with increasing threshold values is seen for higher temperatures. Since the height has been normalized, all the curves in (b) intersect in a same point near H~0.1. This 'isosbestic point is not at H=0 due to the ion-induced asymmetry in the height distribution that bias the renormalization process. The region of the curves > H~0.1 reflect the height of the hydration water and adsorbed Rb^+ ions. Examining the evolution of the surface fraction above H as a function of temperature for given H values > 0.1 exhibits an Arrhenius-type behavior (inset in b). Here we plot, for given threshold values H, the observed surface coverage as a function of the Arrhenius factor. Since most of the coverage is attributed to hydration water, we used the thermally weighed water-mica absorption enthalpy[9-11] (~45 kJ/mol). However, a qualitatively similar results can be achieved if using instead the in the Rb^+ adsorption free energy in water[5,12] ((~20 kJ/mol). Regardless of the threshold value, a same proportionality can be seen between coverage and Arrhenius factor (same slope of the fit, within error).

The characteristic timescales τ_1 and τ_2 calculated from the kinetic traces show little dependence on temperature for a wide range of height thresholds H (c-d). Generally, the timescales tend to decrease with increasing H since higher thresholds emphasize faster adsorbing/desorbing Rb^+ ions over the slower dynamics of hydration water and H_3O^+ in the statistics. For the higher temperatures τ_2 can decrease rapidly past H~0.1, often converging to values close to τ_1 due to the smoother mica surface, making the distinction between the two timescales difficult. An example of analysis is shown in (e) for the data at 35°C with a threshold of 0.1. Both τ_1 and τ_2 are smaller than found in Fig. 2. This is to be expected since this relatively high threshold skews the analysis to lower timescales by strongly limiting the influence of H_3O^+. All the data was acquired over a same experiment and with a single cantilever/tip.

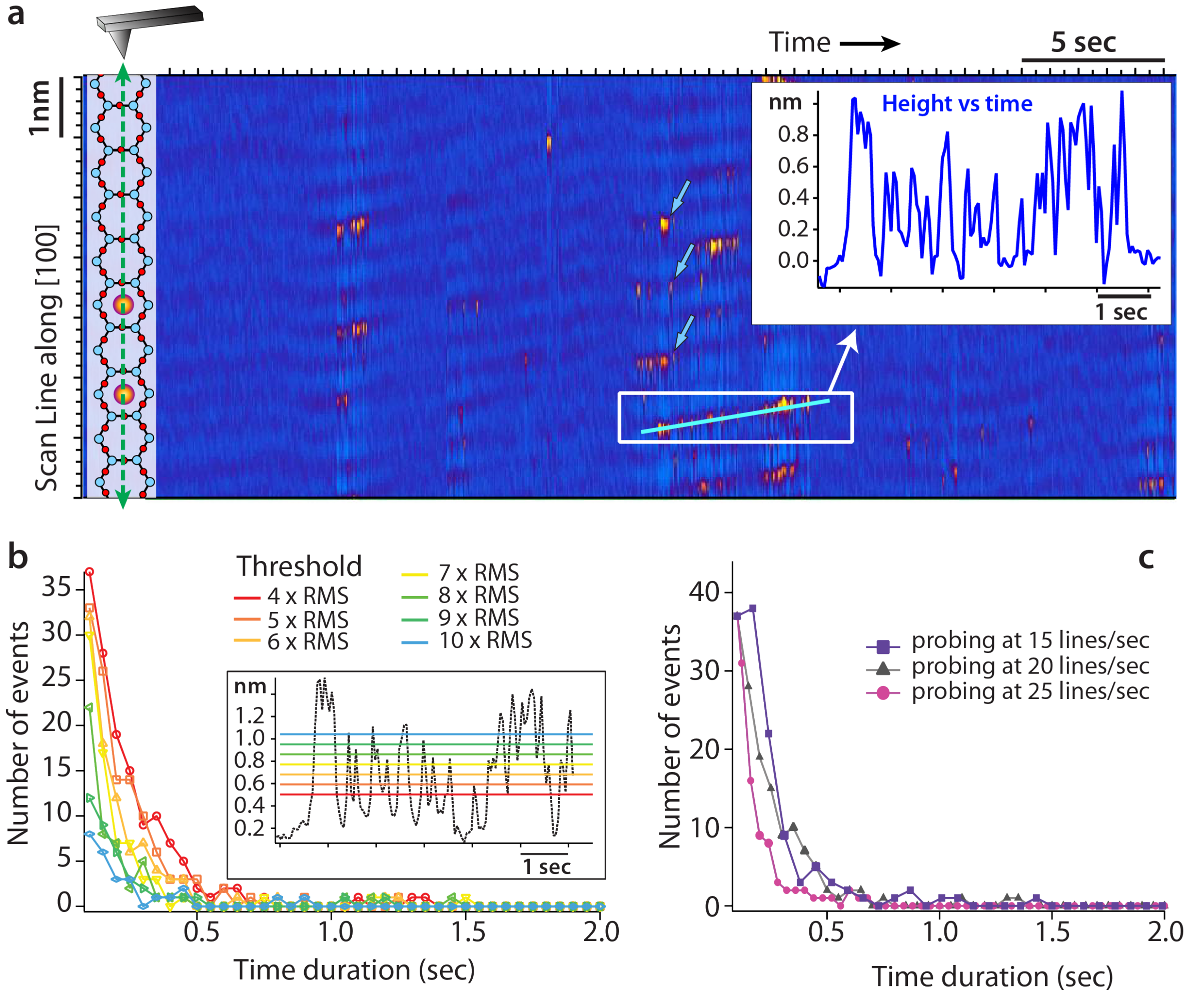

Figure S13: Kinetic experiment conducted with a different AFM and cantilever/tip than used in the rest of this paper (Multimode Nanoscope IIIA, Bruker, Santa Barbara with a cantilever Olympus RC800PSA, Tokyo, Japan). It serves as a control, to ensure that the results are not dependent on the measurement setup. The system is less sensitive and less stable the Cypher and the time resolution is more limited. Kinetic measurements can nonetheless be conducted (a), providing similar results. (a). Due to drift, only short trace fragments can be extracted (inset, a), limiting the thresholding analysis to shorter timescales, as in Fig. 2b. The result show little dependence on the choice of threshold value (b) selected here as multiple of the Root Mean Square value of the trace. The averaged timescale derived is always $\tau 1=115\pm32$ ms (varying from 120 ± 10 ms at $4\times$RMS to 162 ± 5 ms at 10 RMS). Operating at different scan rates (c) yields similar values (between 71 ± 5 ms and 180 ± 20 ms), further confirming the robustness of the result. The study was conducted in 2.5 mM RbCl, ensuring a low apparent surface coverage[5]. As in Fig 2, the scanning direction was set in registry with the mica lattice, along the [100] direction. Part of the image shown in (a) is adapted from Ricci et al[5] (supplementary Fig. 3).

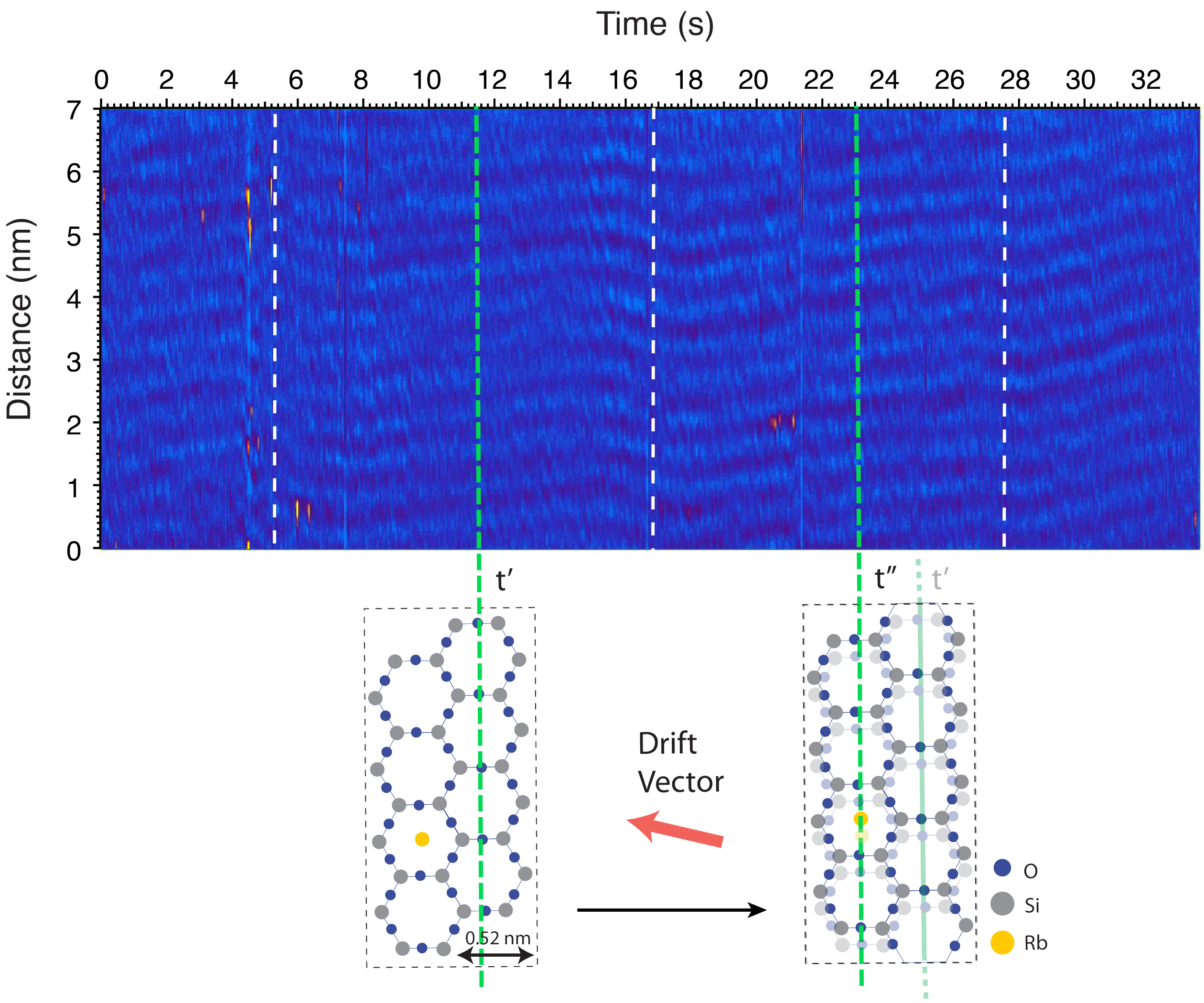

Figure S14: Quantification of the tip drift on the multimode IIIA AFM. An approach identical to that described in Fig. S8 is used: the tip repeatedly scans over the same line. In first approximation, the drift vector (supposed here nearly constant in time and space) has two components: one parallel and one orthogonal to the scan direction (the red arrow in the figure). The drift component parallel to the fast scan direction results in the lattice sites forming oblique lines in time, as opposed to horizontal line. The component perpendicular to the fast scan direction produces an abrupt vertical offset (i.e. an offset parallel to the scanning direction) every ~10 seconds. The magnitude to this offset corresponds to half a lattice site, indicating that the tip has drifted to an adjacent lattice row from its initial position (cartoon). From this interpretation, we can calculate a drift vector with ~80 pm/s parallel to the fast scan direction and ~50 pm/s in the orthogonal direction. The time error resulting from such a drift is negligible in our measurements. Moreover line profiles compensate for the drift parallel to the fast scan direction, and are taken in regions without ‘drift transition’. Note that the scanning rate is about twice slower than with the Cypher AFM (Fig. S8).

Supplementary References

1. Akrami, S. M. R., Nakayachi, H., Watanabe-Nakayama, T., Asakawa, H. & Fukuma, T. Significant improvements in stability and reproducibility of atomic-scale atomic force microscopy in liquid. *Nanotechnology* **25,** 455701 (2014).
2. Voitchovsky, K. High-resolution AFM in liquid: what about the tip? *Nanotechnology* **26,** 100501 (2015).
3. Trewby, W., Livesey, D. & Voitchovsky, K. Buffering agents modify the hydration landscape at charged interfaces. *Soft Matter* **12,** 2642–2651 (2016).
4. Ricci, M., Spijker, P., Stellacci, F., Molinari, J.-F. & Voitchovsky, K. Direct Visualization of Single Ions in the Stern Layer of Calcite. *Langmuir* **29,** 2207–2216 (2013).
5. Ricci, M., Spijker, P. & Voitchovsky, K. Water-induced correlation between single ions imaged at the solid–liquid interface. *Nat. Commun.* **5,** 4400 (2014).
6. Schlegel, M. L. *et al.* Cation sorption on the muscovite (001) surface in chloride solutions using high-resolution X-ray reflectivity. *Geochim. Cosmochim. Acta* **70,** 3549–3565 (2006).
7. Bourg, I. C. & Sposito, G. Molecular dynamics simulations of the electrical double layer on smectite surfaces contacting concentrated mixed electrolyte (NaCl-$CaCl_2$) solutions. *J. Coll. Interf. Sci.* **360,** 701–715 (2011).
8. Meleshyn, A. Potential of Mean Force for Ca^{2+} at the Cleaved Mica−Water Interface. *J. Phys. Chem. C* **113,** 17604–17607 (2009).
9. Delville, A. Monte Carlo Simulations of Surface Hydration: An Application to Clay Wetting. *J. Phys. Chem.* **99,** 2033–2037 (1995).
10. Cantrell & Ewing, G. E. Thin Film Water on Muscovite Mica. *J. Phys. Chem. B* **105,** 5434–5439 (2001).
11. Debbarma, R. & Malani, A. Comparative Study of Water Adsorption on a H^+ and K^+ Ion Exposed Mica Surface: Monte Carlo Simulation Study. *Langmuir* **32,** 1034–1046 (2016).
12. Park, C., Fenter, P. A., Sturchio, N. C. & Nagy, K. L. Thermodynamics, Interfacial Structure, and pH Hysteresis of Rb^+ and Sr^{2+} Adsorption at the Muscovite (001)−Solution Interface. *Langmuir* **24,** 13993–14004 (2008).
